# 3D Printed All‐Natural Hydrogels: Flame‐Retardant Materials Toward Attaining Green Sustainability

**DOI:** 10.1002/advs.202306360

**Published:** 2023-12-14

**Authors:** Xiaoling Zuo, Ying Zhou, Kangan Hao, Chuan Liu, Runhao Yu, Anrong Huang, Chong Wu, Yinye Yang

**Affiliations:** ^1^ College of Materials Science and Engineering Guizhou Minzu University Guiyang 550025 China; ^2^ College of Physics and Mechatronic Engineering Guizhou Minzu University Guiyang 550025 China; ^3^ College of Pharmacy Guizhou University of Traditional Chinese Medicine Guiyang 550025 China; ^4^ National Engineering Research Center for Compounding and Modification of Polymeric Materials Guiyang 550014 China

**Keywords:** 3D printing, biomass‐based hydrogels, flame‐retardant materials, temperature resistance, vat photopolymerization

## Abstract

Biomass‐based hydrogel is a promising flame‐retardant material and has a high potential for applications in transportation, aerospace, building and electrical engineering, and electronics. However, rapid vat photopolymerization (VP) 3D printing of biomass‐based hydrogels, especially that of all‐natural ones, is still rare. Herein, a new class of VP 3D‐printed hydrogels with strong covalent networks, fabricating using fully biomass materials and a commercial liquid crystal display (LCD) printer assembled with low‐intensity visible light is presented. Encouragingly, the highly ordered layer‐by‐layer packing structures provided by VP 3D printing technology endow these hydrogels with remarkable flame retardancy, exceptional temperature resistance, advantageous combustion behaviors, and favorable mechanical strength, in particular, giving them a better limit oxygen index (83.5%) than various biomass‐based hydrogels. The proposed approach enables the green design as well as the precise and efficient preparation for flame‐retardant materials, paving the way for the future flame‐retardant materials toward attaining green sustainability.

## Graphical abstract

The significant plane‐molding offered by vat photopolymerization (VP) 3D printing opens many possibilities for the controllable and efficient manufacturing of biomass materials. A new class of VP 3D‐printed hydrogels with strong covalent networks and highly ordered layer‐by‐layer packing structures, particularly, along with the remarkable flame retardancy, exceptional temperature resistance, advantageous combustion behaviors and favorable mechanical strength, is successfully obtained.

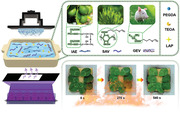

## 1 Introduction

Hydrogels, a class of promising fire‐resistant materials with a water‐rich nature and water‐holding capacity, have received much attention in the fields of coal‐mine fire protection and textile thermal protection.^[^ ^1^, ^2^, ^3^, ^4^, ^5^, ^6^ ^]^ Benefiting from the high heat capacity and latent heat of vaporization of water, hydrogels can excellently perform in terms of fireproofing and extinguishing, because of their extraordinary abilities to reduce the thermal energy and prevent the heat transfer.^[^ ^7^, ^8^ ^]^ The early fire‐retardant hydrogel came into light with the fabrication of cellulose‐based polysaccharides and colloidal silica nanoparticles, which could behave as a carrier for ammonium polyphosphates for wildland fire prevention.^[^ ^9^ ^]^ However, phosphorus has been demonstrated as the priority groundwater contaminant on account of the concern regarding water eutrophication.^[^ ^10^, ^11^ ^]^ Further, the use of petroleum‐based hydrogels also has issues of sustainability and waste pollution, even exacerbates the energy crisis.^[^ ^12^ ^]^ As a result, vigorous exploitation of renewable natural resources is one of the important choices to facilitate the sustainable development of the eco‐environment. It seems that the exploration of environmentally benign flame retardants/flame‐retardant hydrogels is particularly urgent.

Biomass, the largest reservoir of renewable carbon with the component of ≈75% saccharide‐based products, is inherently conducive to constructing flame retardants or flame‐retardant materials due to the bearing of abundant hydroxyl groups.^[^ ^13^ ^]^ From this, various biomass‐based flame retardants (BFR), mainly constituted by saccharide,^[^ ^14^ ^]^ DNA,^[^ ^15^ ^]^ proteins,^[^ ^16^ ^]^ phytic acid^[^ ^17^ ^]^ vegetable oils,^[^ ^18^ ^]^, etc, have been synthesized and extensively developed with their high availability and outstanding char‐forming ability. Moreover, great efforts have also been made in preparing diverse biomass‐based hydrogels (BHG), which mainly consist of physically cross‐linked double networks,^[^ ^19^, ^20^, ^21^ ^]^ semi‐interpenetrating networks,^[^ ^3^, ^7^ ^]^ and organic/inorganic silica hybrid networks.^[^ ^22^ ^]^ However, one thing to note is that the hydrogels developed by blending polymer matrix with BFR generally present the improvement of flame retardation, but meanwhile, followed by the deterioration of comprehensive properties such as thermal stability and mechanical strength, owing to the poor compatibility and dispersibility of BFR with the polymer matrices. The issue of insufficient mechanical properties is likewise inevitable for BHG, ascribable to the weak interactions of non‐covalent bonds. The other thing to note is that the synthesized BFR comes at the price of high‐cost and complex syntheses. In addition, recent preparation methods of BHG have mainly focused on thermal polymerizations, UV irradiation, redox polymerizations, etc that are quite time‐consuming and highly energy‐consuming. These factors impose severe restrictions on the further applications of biomass in the flame‐retardant field. Thus, of particular importance is to establish a green, facile, and efficient strategy for the construction of high‐performance biomass‐based hydrogels.

Among various 3D printing techniques, vat photopolymerization (VP) meets the requirements of low‐energy, high‐performance, and VOC‐free, which has also emerged as a versatile 3D printing technology that features higher geometrical complexity and finer accuracy, with no substantial spatial resolution effect on the printing time.^[^ ^23^, ^24^, ^25^, ^26^, ^27^ ^]^ It is worth noting that liquid crystal display (LCD) 3D printing is particularly prominent because it conquers the phenomenon of pixel distortion as it normally happens with other VP 3D printing techniques,^[^ ^28^ ^]^ such as digital light processing (DLP) printing and stereolithography (SLA) printing. Most importantly, the light shines through the flat LCD panels directly onto the printing inks, leading to the light exposure of a full printing layer, which is of immense benefit to realize the complete plane‐molding in a green and efficient manner.^[^ ^29^ ^]^ But up to now, VP 3D printing of petroleum‐based hydrogels has been demonstrated to be feasible in many works, while the related works carried out for biomass‐based hydrogels are rare, especially for those comprised of hundred‐percent biomass. Because it has been identified that the photopolymerization efficiency of the biomass‐based monomers modified by double bonds is quite low when exposed to UV light.^[^ ^30^ ^]^ It is presumable that the polymerization reactions of biomass‐based monomers should be more difficult under a visible‐light LED lamp with low intensity and even aqueous conditions.

**Figure 1:**
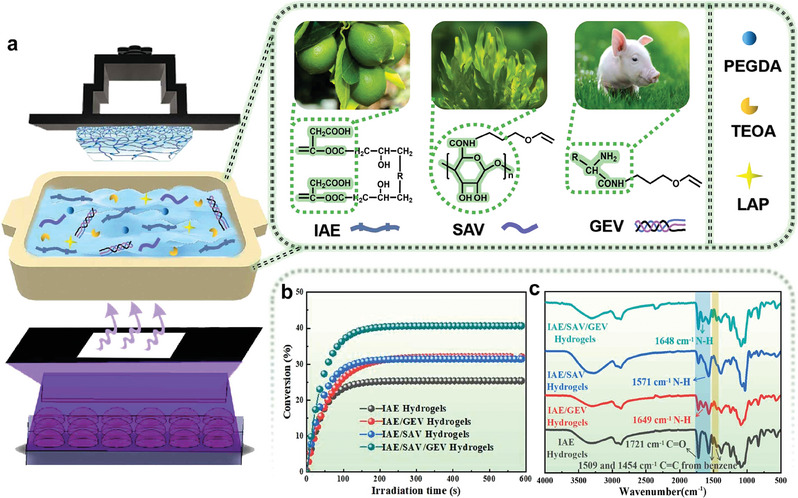
a) LCD 3D printer model and the components of 3D printing inks. b) Alkene function conversions versus time of different 3D printing inks for the fabrication of fully biomass‐based hydrogels in air under 405 nm LED irradiation. c) The FTIR spectra of different freeze‐dried hydrogels.

For these reasons, here we introduce a class of fully biomass‐based printing inks with efficient visible‐light curing capabilities and, subsequently, develop the corresponding hydrogels with excellent flame‐retarded properties and favorable mechanical strength obtained through LCD 3D printing. We successfully merged the intriguing characteristics of the VP 3D printing process with flame retardation and mechanical properties to fabricate all‐natural hydrogels with highly ordered layer‐by‐layer packing structures and strongly cross‐linked covalent networks, along with the exceptional temperature resistance, combustion behaviors, and ultrahigh numerical limiting oxygen index (LOI). These results were achieved by using itaconic acid (IAE)/gelatin‐based (GEV)/sodium alginate (SAV) monomers, while the printing was performed using a commercial LCD printer assembled with a nearly visible LED lamp (405 nm) (**Figure** **1**a). On the background of sustainable development and energy‐efficient strategies, this work offers a general and easily adaptable approach to developing and preparing flame‐retardant materials, for applications in diverse fields, ranging from transportation, aerospace, and building to electronics.

## 2 Results and Discussion

**Figure 2:**
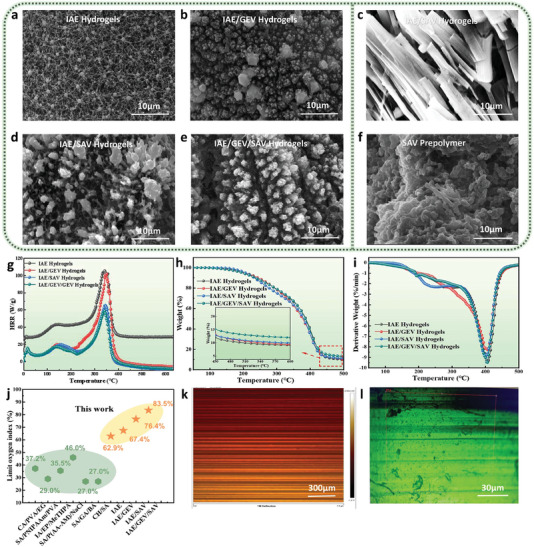
SEM images of the residual chars after LOI tests for a) IAE hydrogels, b) IAE/GEV hydrogels, c,d) IAE/SAV hydrogels and e) IAE/GEV/SAV hydrogels. f) SEM image of the micro‐morphology of SAV monomer. g) Heat release rate (HRR) curves of various hydrogels. h) TGA and i) derivative thermogravimetry (DTG) curves of the dried hydrogels. j) Comparison of the limited oxygen index of all‐natural hydrogels with various biomass‐based hydrogels: calcium alginate/poly(vinyl alcohol)/graphite (CA/PVA/EG),^[^ ^38^ ^]^ sodium alginate/PNIPAAm/polyvinyl alcohol (SA/PNIPAAm/PVA),^[^ ^3^ ^]^ itaconic acid/epoxy resins/methyltetrahydrophthalic anhydride (IA/EP/MeTHPA),^[^ ^39^ ^]^ sodium alginate/poly(acroleic acid‐acrylamide)/sodium chloride (SA/P(AA‐AM)/NaCl),^[^ ^40^ ^]^ sodium alginate/agar/boric acid (SA/GA/BA),^[^ ^41^ ^]^ chitosan/sodium alginate (CH/SA).^[^ ^42^ ^]^ Apparent topography recorded with k) AFM and l) optical microscope (magnification of 40) on the outer surface of IAE/GEV/SAV hydrogels.

At first, we prepared a one‐component printing ink containing IAE and TEOA, and photoinitiator LAP, which presents a double‐bond final conversion (FC) of ≈25.4% after 600 s of 405 nm LED exposure in Figure 1b. Considering that the specially designed monomer IAE is solely soluble in a neutral aqueous solution, so TEOA was employed here as a neutralizer to adjust the pH of the solution. The chemical structure of IAE verified by ^1^H NMR is shown in Figure S1a (Supporting Information). Thanks to the tertiary amine groups, they can also play an effective co‐photoinitiator role in rapidly consuming oxygen and capturing peroxyl radicals, meanwhile, the resulting aminoalkyl radicals are capable of re‐initiating the polymerizations.^[^ ^29^, ^31^ ^]^ The polymerization efficiency can therefore be improved through the inhibition of oxygen impact. From this, the IAE hydrogels were successfully fabricated by VP 3D printing technology via visible light‐triggered aqueous polymerization reaction, confirmed by the absorption peaks of C═O at 1721 cm^−1^ and C═C of benzene at 1454 and 1509 cm^−1^, both assignable to IAE (Figure 1c; Figure S2, Supporting Information). The cross‐linking structures exhibited in Figure S3a (Supporting Information) demonstrate the strong covalent networks existing within IAE hydrogels. In terms of flame performance, these itaconic acid‐based hydrogels reach an LOI value equal to 62.9%, accompanied by a V‐0 rating, the detailed parameters are given in Table S2 (Supporting Information). Their morphology structures after burning were investigated by SEM images. The well‐distributed chars with interconnected multilayer networks are clearly observable in **Figure** **2**a. From Figure 2g, one can see a maximum heat release rate of 105.5 W g^−1^, and, the other lower peak heat release rate (pHRR) is found with a value of 43.3 W g^−1^ in the low‐temperature region. Yet the hydrogels still maintain a constant HRR of around 29 W g^−1^ in the temperature range of 400–600 °C. It follows that there could be room for improvement in the flame retardancy of itaconic acid‐based hydrogels.

Gelatin and sodium alginate, both are naturally occurring polymeric materials and rich in abundant reactive groups (such as amino, carboxyl, and hydroxyl groups), thereby, they have quickly become significant in terms of flame retardancy.^[^ ^3^, ^13^, ^14^ ^]^ It is widely accepted that the high content of carbon and nitrogen leads to their special potentiality of being a charring agent and a blowing agent in fire reactions.^[^ ^32^, ^33^ ^]^ Next we developed two‐component printing inks respectively consisting of IAE and gelatin‐based or sodium alginate‐based monomers, the corresponding chemical structures of GEV and SAV are exhibited in Figure S1b,c (Supporting Information). The IAE/GEV and IAE/SAV printing inks have a similar polymerization efficiency upon visible‐light exposure, allowing a double‐bond FC of 32.1% and 31.5%, respectively (Figure 1b). As shown in Figure 1c and Figure S2 (Supporting Information), the absorption peak at 1636 cm^−1^ is assigned to the amino group of GEV, while it shows a blueshift to 1649 cm^−1^ for IAE/GEV hydrogels. This phenomenon can be attributed to the strong steric hindrance effects caused by the benzene ring of IAE.^[^ ^34^, ^35^ ^]^ Regarding the amino group of SAV, whose absorption peak is red‐shifted from 1600 cm^−1^ to a lower wavenumber of 1571 cm^−1^ for IAE/SAV hydrogels, indicating the intermolecular hydrogen‐bond interactions between IAE and SAV chains.^[^ ^29^ ^]^ Compared with IAE hydrogels, the ever closer cross‐linking structures are obvious in Figure S3b,c (Supporting Information), demonstrating the much stronger covalent networks for the two‐component hydrogels, which correlate well with the increased double‐bond FC values.

Turning to the flammability, the burning ratings of these hydrogels all meet the optimal V‐0 rating. In comparison with IAE hydrogels, an increase in LOI value is achieved for IAE/GEV hydrogels, reaching 67.4%, which is in agreement well with the much denser chars in Figure 2b and the decreased HRR curve in Figure 2g. Concretely, the pHRR values of IAE/GEV hydrogels respectively descend to 19.9 W g^−1^ (139 °C) and 102.1 W g^−1^ (349 °C), meanwhile, the heat release stops at temperatures above 400 °C. As for IAE/SAV hydrogels, whose LOI value greatly rises to 76.4%, and the pHRR values decline to 19.8 (156 °C) and 65.6 (344 °C) W g^−1^. In Figure 2d, the compact chars can be likewise observed after combustion. What is noteworthy is that, in Figure 2c, the residue chars of IAE/SAV hydrogels partially display the flake‐like or strip‐like multilayer substructures, which are closely related to the fibrous state specific to the SAV monomer (Figure 2f). While in Figure S4 (Supporting Information), these unique fibrous structures cannot be observed for the GEV monomer. From these results, we are aware that both SAV and GEV perform well in the improvement of flame retardancy and combustion behaviors for IAE hydrogels, but SAV performs better. It appears that the micro‐morphology of SAV characterized by fibrous structures endows the IAE/SAV hydrogels with superior flame retardancy. Since these multilayer substructures can obstruct the further penetration of heat emission, furthermore, limit the diffusion of oxygen and degradation products, that is, the similar barrier effect derived from other inorganic layered materials.^[^ ^36^, ^37^ ^]^

Relying on the inspiring flame retardancy provided by GEV and SAV, which encouraged to further construct the three‐component printing inks simultaneously containing GEV and SAV to integrate their fire‐retardant characteristics. A double‐bond FC maximum of ≈40.7% is found in Figure 1b, suggestive of the highly efficient photopolymerization reactions of IAE/GEV/SAV printing inks triggered by visible light. The evidence in Figure 1c indicates that the resulting hydrogels emerge the typical absorption peaks of C═O, N─H, and C═C of benzene, identical to the two‐component hydrogels. The most closer cross‐linking structures displayed in Figure S3d (Supporting Information) suggest the strongest covalent networks among these hydrogels. What is more, the LOI value of IAE/GEV/SAV hydrogels greatly increases to 83.5%, and the UL‐94 rating still remains the same (V‐0 rating). The residues after combustion, presented in Figure 2e, show homogeneous and more compact chars than those of two‐component hydrogels, consistent well with the increased number of residues collected after TGA tests (Figure 2h–i; Table S3, Supporting Information). Concretely, the number of residues for IAE, IAE/GEV, and IAE/SAV hydrogels is roughly equivalent to 9.1%, 9.4% and 9.9%, lower than that of IAE/GEV/SAV hydrogels, which reaches a value of 12.0%. Besides, the thermal stability investigated for these hydrogels presents that the values of maximum degradation temperature (T_max_) gather in a narrow range of 402–407 °C (listed in Table S3, Supporting Information), manifesting their fine thermal stability.

With regard to combustion behaviors, a lower pHRR is found with a value of 59.6 W g^−1^ at 341 °C, the other one appears at 139 °C with 17.4 W g^−1^ (Figure 2g; Table S2, Supporting Information). Compared with the published work, in general, the as‐prepared biomass‐based hydrogels were designed to cross‐link with at least one of our three components.^[^ ^3^, ^38^, ^39^, ^40^, ^41^, ^42^ ^]^ Our fully biomass‐based hydrogels have significant advantages in limiting oxygen index, implying their outstanding flame performance (Figure 2j). This beneficial phenomenon can be attributed not only to the synergistic flame‐retarded effects of IAE, GEV, and SAV components but also to the peculiar manufacturing pattern: LCD 3D printing. The highly ordered layer‐by‐layer packing structures obtained in Figure 2k,l also contribute to effectively realizing the heat and oxygen barrier performance. As a comparison, the IAE/GEV/SAV hydrogels, prepared by 405 nm LED irradiation rather than LCD 3D printing with the same exposure time, although they maintain the optimal V‐0 rating but own a LOI value of 72.6%, far less than 83.5%. It is obvious that LCD 3D printing, a bottom‐up strategy, can provide space for the structural design and fabrication of flame‐retardant materials.

**Figure 3:**
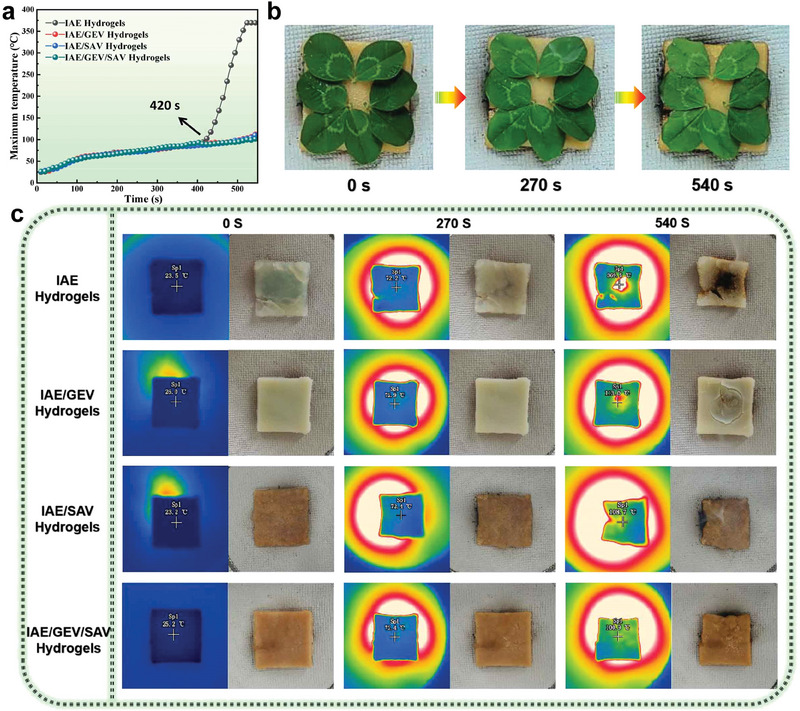
a) Time‐dependent temperature profiles of the different hydrogel surfaces. b) Images of fresh four‐leaf clovers subjected on the alcohol burner for 9 min. c) Infrared and digital images of the different hydrogels during the 0 s, 270 s and 540 s of heating.

To better illustrate the thermal insulation of 3D‐printed all‐natural hydrogels, a flame temperature of ≈600 °C was placed under the hydrogels, and the space between them was fixed at 50 mm. The hydrogels were prepared with a length of 50 mm, width of 50 mm, and thickness of 5 mm. Infrared (IR) images of the hydrogels were tested by an infrared imager (Fortric 226S) every 5 s, the center temperature of hydrogel surfaces with increasing time is depicted in **Figure** **3**a. For all hydrogels, they hold a similar temperature (below 100 °C) before 420 s. After that, IAE hydrogels drastically rise with time and reach 370 °C after 540 s of heating. While the others gently increase with the heating time, in detail, IAE/GEV and IAE/SAV hydrogels ascend to 113 and 109 °C after heating, the surface temperature of IAE/GEV/SAV hydrogels is the lowest, only 101 °C, slightly higher than the boiling temperature of the water. Therefore, the IR and photos in Figure 3c show that all hydrogels nearly keep the original appearance after 270 s of heating. Nevertheless, the central part of IAE hydrogels is almost burnt after 540 s of heating, suggesting that the hydrocarbon structure of IAE chains is severely destroyed by flaming. While for other hydrogels, volume shrinkage caused by the release of physically and chemically bonded water,^[^ ^43^ ^]^ together with the mild to moderate destruction of cross‐linking networks, are the most obvious features. It can be determined that all the fully biomass‐based hydrogels exhibit splendid thermal insulating ability, nothing but IAE/GEV/SAV hydrogels do the best. These results are closely associated not only with the heat‐absorbing action specific to water,^[^ ^7^, ^8^ ^]^ but also with the most closely cross‐linked networks of IAE/GEV/SAV hydrogels, which yield the high‐performance heat resistance to delay the degradation of polymer chains and preserve the excellent thermal insulation.^[^ ^44^ ^]^ More interestingly, the fresh four‐leaf clovers could be well protected by IAE/GEV/SAV hydrogels for at least 9 min without withering or carbonization under the heat of an alcohol burner (Figure 3b).

**Figure 4:**
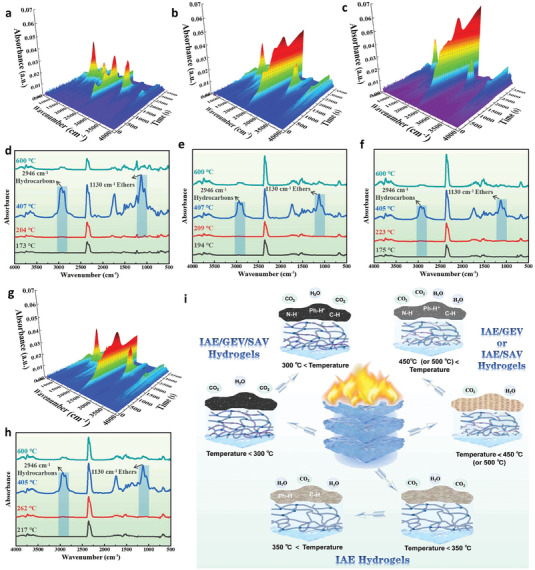
FTIR spectra of the volatile gases from a–d) IAE hydrogels, b,e) IAE/GEV hydrogels, c,f) IAE/SAV hydrogels and g,h) IAE/GEV/SAV hydrogels under N_2_ atmosphere. i) The possible burning processes for the different hydrogels.

TG‐IR tests were conducted to study the release of gaseous products for all hydrogels during thermal decomposition, with the curves shown in **Figure** **4**a–g. Their main decomposition products are similar: in particular, water (3737 cm^−1^), hydrocarbons (2946 cm^−1^), carbon dioxide (2360 cm^−1^), carbonyl compounds (1736 cm^−1^), and ethers (1130 cm^−1^).^[^ ^45^ ^]^ Importantly, the introduction of GEV and SAV into IAE hydrogels postpones the occurrence time of hydrocarbons and ethers (Figure 4d–h). For example, the characteristic absorption peaks of hydrocarbons and ethers produced by IAE hydrogels appear at 173 and 204 °C, respectively (Figure 4d), earlier than those of other hydrogels (Figure 4e–h). Especially for IAE/GEV/SAV hydrogels, which produce the hydrocarbons and ethers at 217 and 262 °C, respectively (Figure 4h). This fact suggests that GEV and SAV suppress the thermal decomposition of IAE matrix. Further, the nonflammable gases, i.e., H_2_O and CO_2_, produced by IAE/GEV/SAV hydrogels, are slightly above those of IAE hydrogels, but far less than those of the two‐component hydrogels (Figure 4a–g; Figure S5, Supporting Information). This result reveals that the gaseous phase activity of GEV or SAV using alone exerts more effects than that of GEV and SAV combinations to promote the flame retardancy of IAE hydrogels.

The FTIR spectra that the condensed phase products of all hydrogels were calcined in the muffle at different temperatures are shown in Figure S6 (Supporting Information). The C═O of carboxyl groups (1721 cm^−1^) can be completely consumed when the temperature increases to 350, 500, and 450 °C for IAE hydrogels, IAE/GEV, and IAE/SAV hydrogels, respectively, via decarboxylation pattern.^[^ ^32^ ^]^ While this characteristic peak is consumed within IAE/GEV/SAV hydrogels as early as 300 °C, the chars are accordingly generated to hinder the further decomposition of the matrix, consequently, resulting in the decreased release of nonflammable gases. The characteristic band around 1167 cm^−1^ represents C─O─C obtained through the esterification pathway,^[^ ^33^ ^]^ which is generated in the temperature range of 400–600 °C for IAE hydrogels, and 300–450 °C for both IAE/GEV and IAE/SAV hydrogels, but which cannot be detected for IAE/GEV/SAV hydrogels, partially accounting for the generation of more water for the two‐component hydrogels compared with IAE/GEV/SAV hydrogels.

Based on the above results, it is concluded that the synergistic mechanisms of the gaseous phase and condensed phase endow the fully biomass‐based hydrogels with remarkable flame‐retardant properties. But the occurrence time of gaseous‐phase flame retardant behavior differs from that of condensed‐phase flame retardant behavior for different hydrogels. The burning processes described in Figure 4i presents that, in the temperature range below 450 °C (for IAE/SAV hydrogels) or 500 °C(for IAE/GEV hydrogels), the introduction of GEV or SAV into IAE hydrogels greatly improves the release of noncombustible gases (e.g., CO_2_ and H_2_O) to dilute the concentration of combustible gases. After that, the compact char residue can be totally formed, along with the more severe release of CO_2_, to synergistically protect the matrix from further degradation. In the temperature range below 350 °C (IAE hydrogels) or 300 °C (IAE/GEV/SAV hydrogels), the formation of chars plays the dominant role in resisting the fire penetration, in particular, which is more efficient for IAE/GEV/SAV hydrogels. However, the gaseous phase activity of IAE/GEV/SAV hydrogels is less effective relative to the two‐component hydrogels.

**Figure 5:**
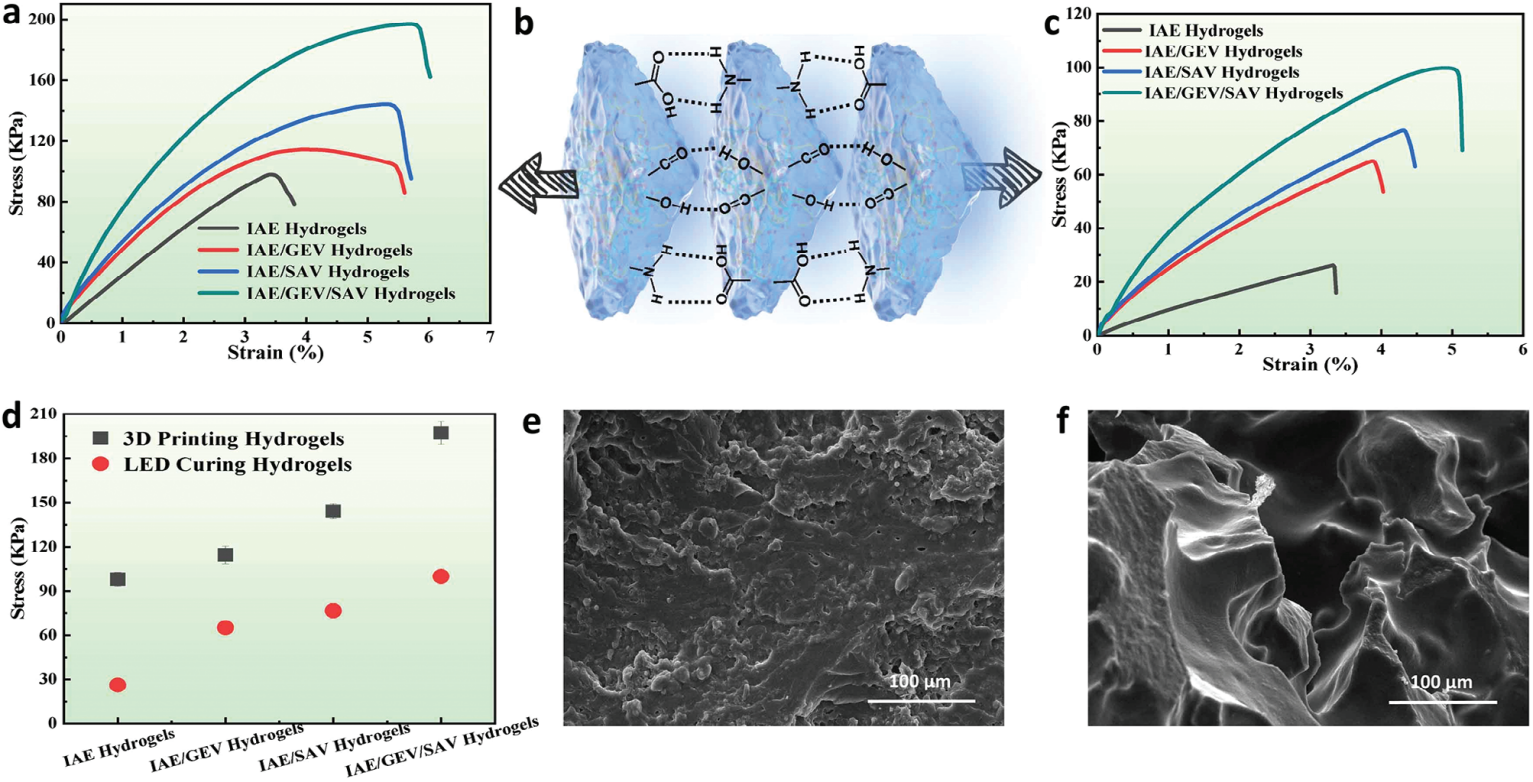
Tensile stress‐strain curves for a) LCD 3D printed hydrogels and c) 405 nm LED cured hydrogels (the same exposure time with 3D printing). b) Schematic illustration of 3D printed IAE/GEV/SAV hydrogels bearing load. d) Comparison of stress values between 3D printed hydrogels and LED cured hydrogels. SEM images of the fracture morphology for e) 3D printed hydrogels and f) 405 nm LED cured hydrogels.

Pure gelatin and sodium alginate hydrogels are generally considered to have poor mechanical properties.^[^ ^32^, ^46^, ^47^ ^]^ Nevertheless, the employment of VP 3D printing technique is in favor of obtaining the all‐natural hydrogels with satisfactory mechanical properties. As expected, inspiring results of all hydrogels were achieved from static mechanical tests, with the decent stress‐strain curves shown in **Figure** **5**a and the data collected in Tables S4 (Supporting Information). Clearly, the stress increases with the introduction of GEV and SAV into IAE hydrogels, is measured respectively as 114.5 (IAE/GEV hydrogels) and 144.3 KPa (IAE/SAV hydrogels) versus 98.0 KPa (IAE hydrogels). Relative to IAE/GEV hydrogels, the higher tensile strength specific to IAE/SAV hydrogels can be attributable to the existence of intermolecular hydrogen‐bond interactions, as evidenced in Figure 1c and Figure S2 (Supporting Information). Thankfully, the combination of three monomers exerts the stress of IAE/GEV/SAV hydrogels up to 197.1 KPa. Up to now, the excellent mechanical properties of the reported all‐natural hydrogels mostly rely on inherently high‐strength constituents, such as cellulose nanocrystals or nanoclay.^[^ ^4^, ^48^ ^]^ After all, there are few reports of all‐natural hydrogels composed of inherently weak‐strength constituents, i.e., itaconic acid, gelatin, and sodium alginate used in this work, particularly related to those consisted of covalently cross‐linked structures and with well‐strength. Remarkably, as depicted in Figure 5b, due to the highly ordered layer‐by‐layer packing structures, which provide high packing density and inter‐bonding strength to dissipate stress and resist damage.^[^ ^49^ ^]^ Hence, the tensile stress of 3D‐printed hydrogels is far greater than that of LED‐cured hydrogels (Figure c,d). Additionally, the fracture surface with more flat and neat morphology shown in Figure 5e indicates that the bearing condition of 3D printed hydrogels is relatively uniform compared with that of LED cured hydrogels. The fracture surface is severely rough and irregular in Figure 5f, suggestive of the heterogeneity of cross‐linking structures within LED cured hydrogels. These inspiring results make the 3D printed all‐natural hydrogels a potential sustainable flame‐retardant material possessing favorable mechanical strength for a broader application in different scientific areas, especially in the burgeoning flexible electronics field.

## 3 Conclusion 

The significant plane‐molding offered by LCD 3D printing opens many possibilities for the controllable and efficient manufacturing of biomass materials. Taking advantage of this technology, we have successfully fabricated a class of fully biomass‐based flame‐retardant hydrogels from the renewable itaconic acid, gelatin and sodium alginate via strong covalent cross‐linking. The highly ordered layer‐by‐layer packing structures ensure a combination of excellent temperature resistance, advantageous combustion behaviors and favorable mechanical strength for the fully biomass‐based hydrogels. Notably, these potential sustainable materials achieve the outstanding flame‐resisting performance with an optimal limiting oxygen index of 83.5%, which is superior to the previously reported biomass‐based hydrogels. Overall, this work not only reports a class of all‐natural hydrogels fabricated by LCD 3D printing technology when coupled with their exceptional functional performance, hold promising potential for many important industrial applications. In addition, this work but also provides a new perspective on the design and preparation for the future flame‐retardant materials in a green, efficient and eco‐friendly way. Other works about their more properties, e.g., self‐healing, anti‐freezing and long‐term moisture properties are under progress.

## Conflict of Interest

The authors declare no conflict of interest.

## Supporting information

Supporting Information

## Data Availability

The data that support the findings of this study are available from the corresponding author upon reasonable request.
