# Supplementary material for: 3D Printed All‐Natural Hydrogels: Flame‐Retardant Materials Toward Attaining Green Sustainability
Source: Adv Sci (Weinh). 2023 Dec 14;11(3):2306360. doi: 10.1002/advs.202306360 (PMC10797461; doi:10.1002/advs.202306360)
Supplement: Supplementary file 1 — Supporting Information [file ADVS-11-2306360-s001.pdf]

ADVANCED SCIENCE
Open Access

Supporting Information

for *Adv. Sci.*, DOI 10.1002/advs.202306360

3D Printed All-Natural Hydrogels: Flame-Retardant Materials Toward Attaining Green Sustainability

Xiaoling Zuo, Ying Zhou, Kangan Hao, Chuan Liu, Runhao Yu, Anrong Huang, Chong Wu* and Yinye Yang**

Supplementary Materials for

3D Printed All-Natural Hydrogels: Flame-Retardant Materials Toward Attaining Green Sustainability

Xiaoling Zuo[#*], Ying Zhou[#], Kangan Hao, Chuan Liu, Runhao Yu, Anrong Huang, Chong Wu[*], Yinye Yang[*]

Corresponding Author:

*E-mail: shanghai0401@163.com (Xiaoling Zuo), wuchong_chem@163.com (Chong Wu), Ythin1969@sohu.com (Yinye Yang)

The PDF file includes:

- Scheme S1. The synthetic route of IAE.
- Scheme S2. The synthetic route of SAV.
- Scheme S3. The synthetic route of GEV.
- Table S1. The compositions of various fully biomass-based hydrogels.
- Figure S1. The ^{1}H NMR spectra of (a) IAE in CD_4O, (b) GEV and (c) SAV in D_2O.
- Figure S2. The FTIR spectra of pure IAE, GEV and SAV monomers.
- Figure S3. SEM image of the surface of the dried hydrogels. (a) IAE hydrogels, (b) IAE/GEV hydrogels, (c) IAE/SAV hydrogels and (d) IAE/GEV/SAV hydrogels.
- Table S2. Results from LOI, UL-94 and MCC for different hydrogels.
- Figure S4. SEM images of the micro-morphology of GEV monomer.
- Table S3. The key data obtained from TGA tests for all hydrogels.
- Figure S5. FTIR spectra of the pyrolysis products of all hydrogels at T_{max}.
- Figure S6. FTIR spectra of the the condensed products of (a) IAE hydrogels, (b) IAE/GEV hydrogels, (c) IAE/SAV hydrogels and (d) IAE/GEV/SAV hydrogels at different temperatures.
- Table S4. Stress values for 3D printed hydrogels and 405 nm LED cured hydrogels.

Supplementary Materials

S1. Supplementary Materials and Characterization

Materials

Itaconic acid (IA, 99%) was purchased from TCI Co., Ltd. Poly(ethylene glycol) diacrylate (PEGDA, M_w 700), sodium alginate (SA, 99%), gelatin (GE, 99%) and poly(ethylene glycol) diglycidyl ether (PEGGE, 99%) were purchased from Macklin Biochemical Co., Ltd. Triethanolamine (TEOA, 99%) was purchased from Aladdin Chemistry Co., Ltd. 4-Methoxyphenol (99%), ethanol (95%), N-hydroxysuccinimide (NHS, 98%), 1-(3-dimethylaminopropyl)-3-ethylcarbodiimide hydrochloride (EDC, 99%) and N,N-dimethylbenzylamine (99%) were obtained from Innochem Co., Ltd. 2-Hydroxyethyl methacrylate (HEMA, water≤50 ppm, 99%) was gotten from Adamas Co., Ltd. Epoxide resin E-51 (EP, epoxide number 0.48-0.52) was supplied by Mreda Co., Ltd. Diethyl ether (98%) and hydrochloric acid (98%) were purchased from Sinopharm Chemical Reagent Co., Ltd. Lithium phenyl(2,4,6-trimethylbenzoyl)phosphin (LAP, 97%) and 3-(vinyloxy)propan-1-amine (APVE, 97%) were obtained from Leyan Co., Ltd. All the reagents and solvents were used as received without further purification.

Characterization

Characterization of structural features and polymerization kinetics

Proton nuclear magnetic resonance spectroscopy (^1H NMR) spectra of IAE, SAV and GEV were recorded on a Bruker Advance AMX-400 Spectrometer in CD_4O and D_2O, respectively.

The evolution of the double bond content of 3D printing inks was continuously followed by real-time fourier transform infrared reflection (RT-FTIR) spectroscopy (JASCO FTIR 4100) in the range of 870-970 cm^{-1}. Polymerization kinetics were assessed *in situ* by integrating the attenuance of the double-bond vibrational bands during 405 nm LED irradiation.[1] The infrared spectra of the hydrogels in dry state were recorded by attenuated total reflectance Fourier transform infrared spectroscopy. While the variations of the chemical structures of residue chars heated by different temperatures (10 min) were recorded by Fourier transform infrared spectroscopy using KBr pellets. During the testing process, the samples quality had a constant value, and the quality proportion of the samples

to KBr was fixed.[2] The spectra were obtained with 16 scans and a resolution of 4 cm^{-1} in the range of 4000-500 cm^{-1} at room temperature.

Characterization of flame retardancy and combustibility

Firstly, the hydrogels were placed in a constant temperature and humidity chamber (25 °C, relative humidity (RH) 65 %) for 24 h before testing. The flame retardancy of all hydrogels was evaluated by limiting oxygen index (LOI) and UL-94 tests. The LOI value was surveyed on a JF-3 oxygen index meter (Jiangning, China) with the dimensions of 80 × 10 × 4 mm^3 in accordance with the ASTM D2863-2006.[3] LOI analysis was tested three times repeatedly to obtain the average values in this work. The UL-94 test was performed according to ASTMD 3801 on a SH5300 instrument (Guangzhou, China). The specimens used for this test were of the dimensions of 130 × 13 × 3.2 mm^3.[4]

The micro-scale combustion calorimetry (MCC) test was carried out on FAA-PCFC (Fire Testing Technology Limited) according to ASTM D7309-21a under a relative humidity 50 ± 5% at 25 ± 2 °C.[5] The LOI and MCC values were the average of three measurements and the results were considered to be reproducible to ± 10%.

Characterization of morphology features

SEM images obtained on KYKY-2800B (KYKY Technology Development, China) were used to investigate the residue chars after LOI tests for all hydrogels with an acceleration voltage of 20 KV. All samples were recorded after gold coating surface treating.

Firstly, the hydrogels were placed in a constant temperature and humidity chamber (25 °C, relative humidity (RH) 65 %) for 24 h before testing. AFM measurements were obtained using a Bruker Dimension Icon AFM. Data analysis and image processing were conducted with NanoScope Analysis software (version 1.85) and JPK SPM Data Processing 8.0.13.

Characterization of thermal stability and decomposition

Thermogravimetric analysis/infrared spectrometry (TG-IR) spectra of all hydrogels in dry state were studied by a TG analyzer (STA 449 C, Germany) equipped with an infrared spectrometer (Tensor 27, Germany) from room temperature to 600 °C at a heating rate of 5 °C/min under nitrogen atmosphere to investigate the gas components resulting from thermal decomposition. About 10 mg of each of the sample was loaded in a platinum

sample pan. The TGA values were the average of three measurements and the results were considered to be reproducible to ± 10%.

Characterization of mechanical strength

Tensile stress-strain tests were carried out with a commercial electronic universal tensile stress tester (WDW-10) with a 50 N load cell, in accordance with GB/T1040-2006 standards. The hydrogels were printed into standard dumbbell shapes with 2 mm thickness, 5 mm width, and 70 mm gauge length. The original hydrogels were kept in chambers with certain relative humidity and temperature till they reached their steady states. Then uniaxial tensile tests were performed immediately at a rate of 20 mm/min using a tensile machine. Each parameter was the average of five measurements.

S2. Chemical modification of itaconic acid

Itaconic acid (2.248 g), N,N-dimethylbenzylamine (0.021 g) and 4-methoxyphenol (0.023 g) were dissolved in the co-solvent, 2-hydroxyethyl methacrylate (2.5 g), and then the solution was heated and stirred at 80 ℃. The mixture of epoxide resin (2.1 g) and poly(ethylene glycol) diglycidyl ether (2.47 g) was added into the aforementhioned solution within 0.5 h after stirring for 3 h at room temperature. Subsequently, the solution was stirred and heated at 105 °C for 3 h. The liquid products with light yellow IAE were obtained after the reaction.

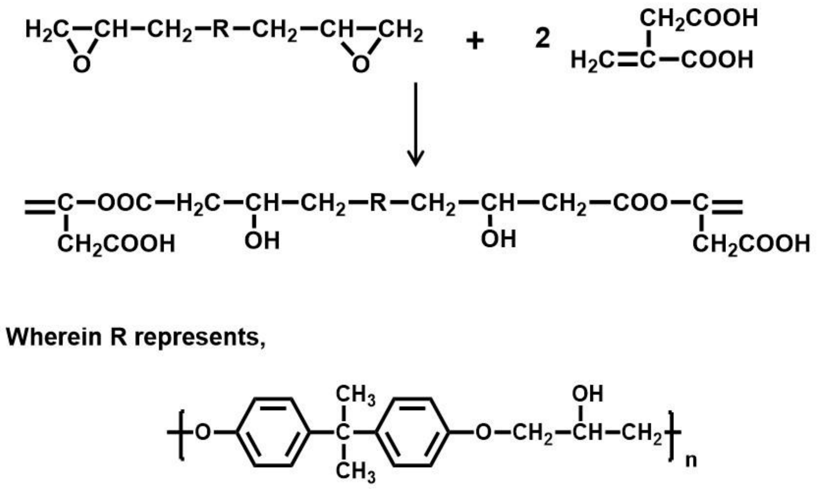

Scheme S1. The synthetic route of IAE.

S3. Chemical modification of sodium alginate

Sodium alginate was chemically modified according to the reported protocol.[6] Sodium alginate (0.5 g) was dissolved in HCl aqueous solution (50 mL, pH = 4) under magnetic stirring at room temperature. And then, 1-(3-dimethylaminopropyl)-3-ethylcarbodiimide hydrochloride (0.47 g) and N-hydroxysuccinimide (0.282 g) were added as catalysts and stirred for 3 h. Subsequently, 0.835 mL of 3-(vinyloxy)propan-1-amine was added drop-wisely, the obtained mixture continued stirring for 16 h in dark, followed by precipitation in excessive ethanol. The final products were washed by 5 times with ethanol and 3 times with diethyl ether. The residual solvents were removed by rotary evaporator to obtain the crude product. The white fibrous products SAV were kept under 4 °C in dark.

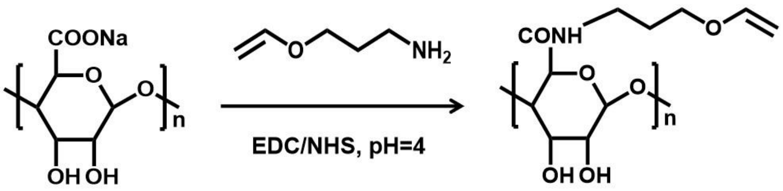

Scheme S2. The synthetic route of SAV.

S4. Chemical modification of gelatin

Gelatin (0.5 g) was dissolved in 100 mL aqueous solution under magnetic stirring at 35 °C. And then, 1-(3-dimethylaminopropyl)-3-ethylcarbodiimide hydrochloride (0.314 g) and N-hydroxysuccinimide (0.188 g) were added as catalysts and stirred for 3 h. Subsequently, 0.557 mL of 3-(vinyloxy)propan-1-amine was added drop-wisely after heating the solution, the obtained mixture continued stirring for 18 h in dark, followed by precipitation in excessive ethanol. The final products were washed by 5 times with ethanol and 3 times with diethyl ether. The residual solvents were removed by rotary evaporator to obtain the crude product. The cream-colored products GEV were kept under 4 °C in dark.

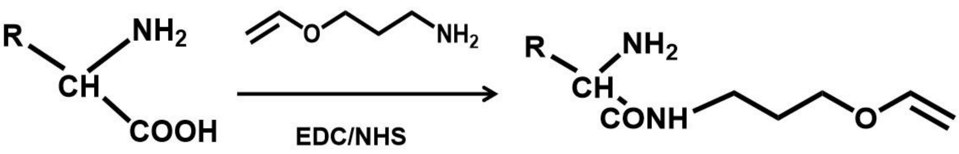

Scheme S3. The synthetic route of GEV.

S5. 3D printing of various hydrogels

The IAE hydrogels were prepared by an aqueous solution containing IAE and TEOA with photoinitiator LAP. The IAE/SAV hydrogels were prepared by an aqueous solution

containing IAE, SAV and TEOA with a cross-linking monomer PEGDA and photoinitiator LAP, while the IAE/GEV hydrogels were prepared by the same formulation in the absence of SAV but in the presence of GEV. The IAE/GEV/SAV hydrogels were prepared by an aqueous solution containing IAE, SAV, GEV and TEOA with a cross-linking monomer PEGDA and photoinitiator LAP. The detailed weight of each composition is listed in Table S1. Afterward, the printing inks for IAE and IAE/SAV hydrogels were thoroughly stirred at room temperature for 24 h, while the printing inks for IAE/GEV and IAE/GEV/SAV hydrogels hydrogels were thoroughly stirred at 35 °C for 24 h. Note that TEOA plays a role of neutralizer in adjusting the pH to 7.

A predesigned cuboid hydrogel was 3D printed using a LCD 3D printer (LD-006, Creality) with a 405 nm LED light source (λ: 375-435 nm, irradiance = 14.5 mW·cm^{-2}). This printer was operated by a top-down LCD system with a digital mirror device. A designed 3D model was first sliced into a series of 2D images. During the printing, the parameters of base layers and connected layers were set to 10 and 12, respectively. Regarding IAE hydeogels, the sliced thickness of each layer was set to 30 μm, the exposure time was set to approximately 8 s for the first layer, and the exposed time of each layer was 7 s. For IAE/GEV hydeogels, the sliced thickness of each layer was set to 30 μm, the exposure time was set to approximately 5 s for the first layer, and the exposed time of each layer was 5 s. While for IAE/SAV and IAE/GEV/SAV hydrogels, the sliced thickness of each layer was set to 30 μm, the exposure time was set to approximately 9 s for the first layer, and the exposed time of each layer was 10 s. After printing, the obtained hydrogels were rinsed with water in a sonicator bath for 2 min to remove the monomers/photoinitiator residues and additives. The printed hydrogels were stored in a sealed environment with a high moisture content to prevent excessive water evaporation.

Table S1. The compositions of various fully biomass-based hydrogels.

Weight (g)	DIW	IAE	GEV	SAV	PEGDA	LAP	TEOA
IAE hydrogels	50	20	-	-	-	0.6	4
IAE/GEV hydrogels	50	20	5	-	0.25	0.75	4
IAE/SAV hydrogels	50	20	-	5	0.25	0.75	4
IAE/GEV/SAV hydrogels	50	20	5	5	0.3	0.9	4

S6. Supplementary Figures and Tables

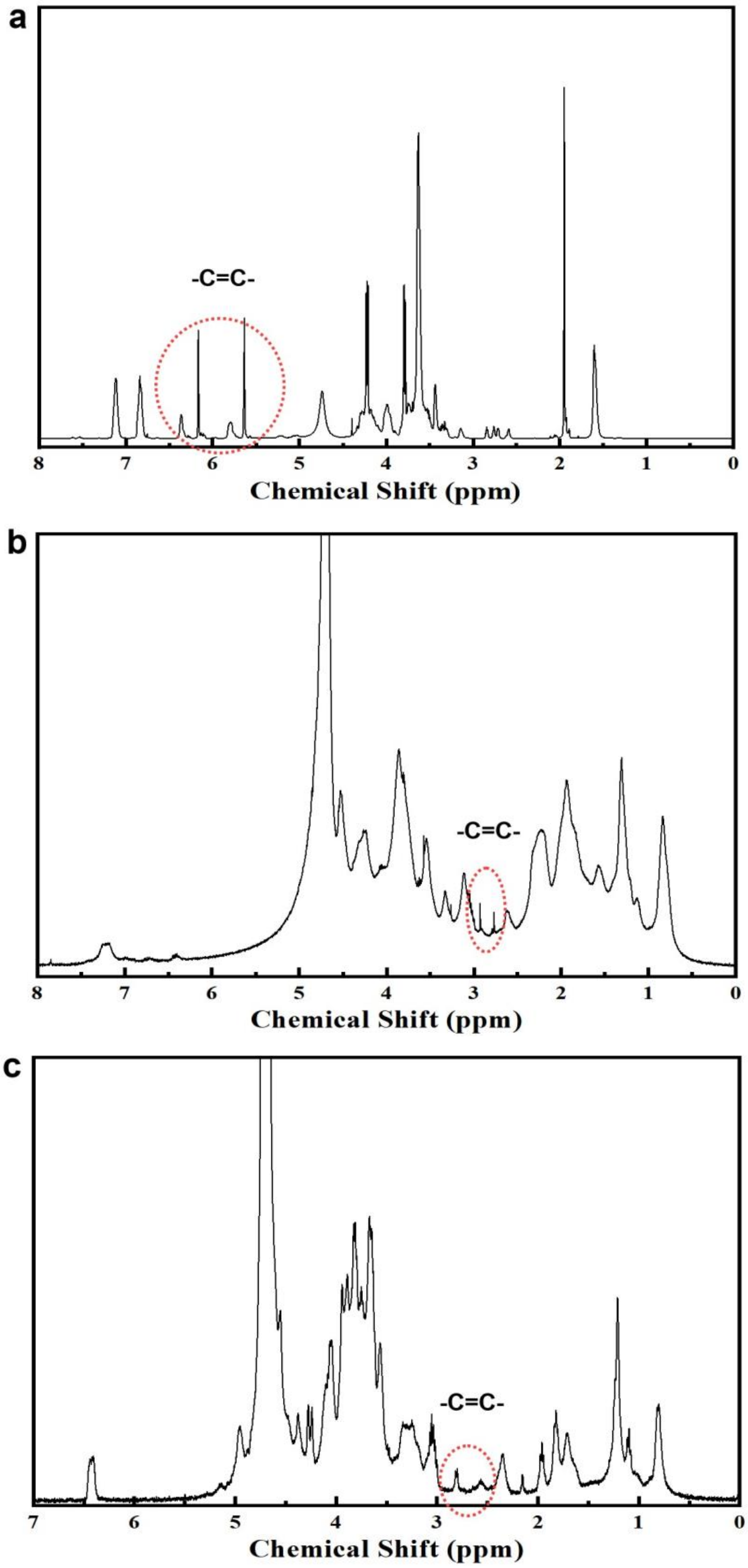

Figure S1. The ^{1}H NMR spectra of (a) IAE in CD_4O, (b) GEV and (c) SAV in D_2O.

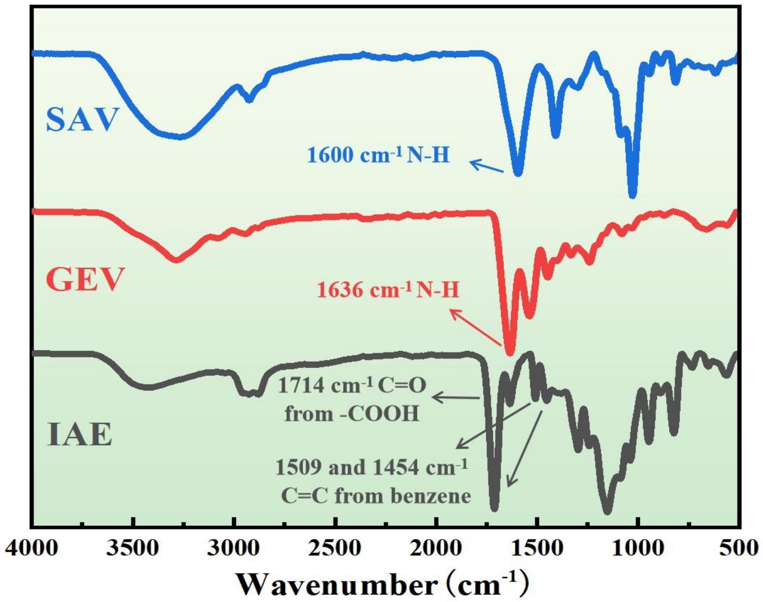

Figure S2. The FTIR spectra of pure IAE, GEV and SAV monomers.

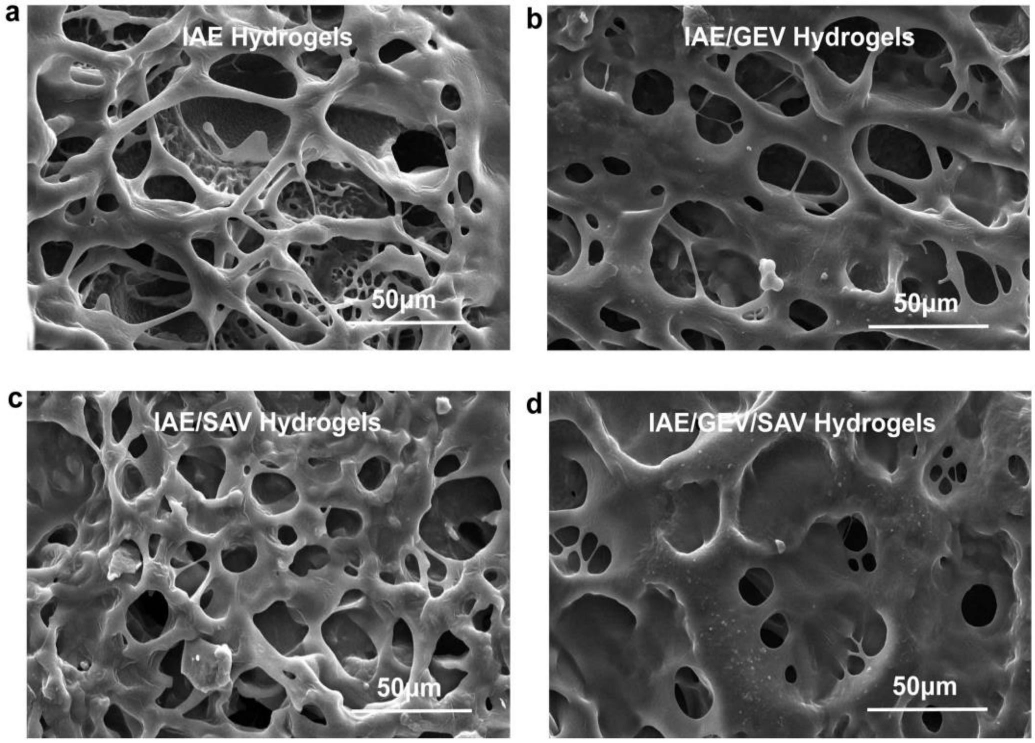

Figure S3. SEM image of the surface of the dried hydrogels. (a) IAE hydrogels, (b) IAE/GEV hydrogels, (c) IAE/SAV hydrogels and (d) IAE/GEV/SAV hydrogels.

Table S2. Results from LOI, UL-94 and MCC for different hydrogels.

Weight (g)	LOI (%)	UL-94	pHRR-1		pHRR-2	
			T (°C)	Value (W/g)	T (°C)	Value (W/g)
IAE hydrogels	62.9 ± 0.6	V-0	342 ± 1.0	105.5 ± 0.4	140 ± 1.1	43.3 ± 0.3
IAE/GEV hydrogels	67.4 ± 0.4	V-0	349 ± 0.8	102.1 ± 0.5	139 ± 1.5	19.9 ± 0.4
IAE/SAV hydrogels	76.4 ± 0.3	V-0	344 ± 1.2	65.6 ± 0.4	156 ± 0.9	19.8 ± 0.4
IAE/GEV/SAV hydrogels	83.5 ± 0.5	V-0	341 ± 1.4	59.6 ± 0.6	139 ± 1.4	17.4 ± 0.5
IAE/GEV/SAV hydrogels (prepared by 405 nm LED irradiation)	72.6± 0.6	V-0	-	-	-	-

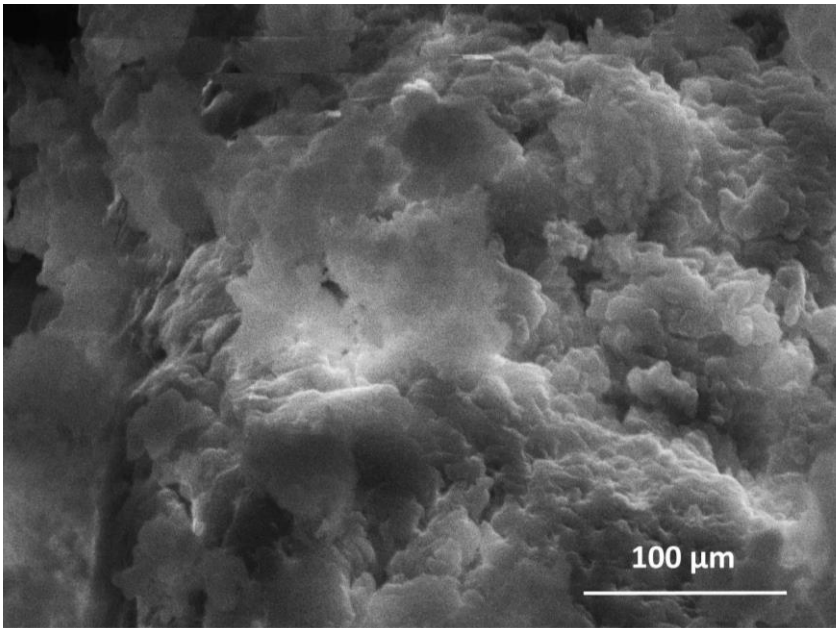

Figure S4. SEM images of the micro-morphology of GEV monomer.

Table S3. The key data obtained from TGA tests for all hydrogels.

Weight (g)	T_{max} (℃)	Char at 600 ℃ (%)
IAE hydrogels	407 ± 1.5	9.1 ± 0.1
IAE/GEV hydrogels	407 ± 2.1	9.4 ± 0.2
IAE/SAV hydrogels	402 ± 2.7	9.9 ± 0.2
IAE/GEV/SAV hydrogels	405 ± 1.9	12.0 ± 0.1

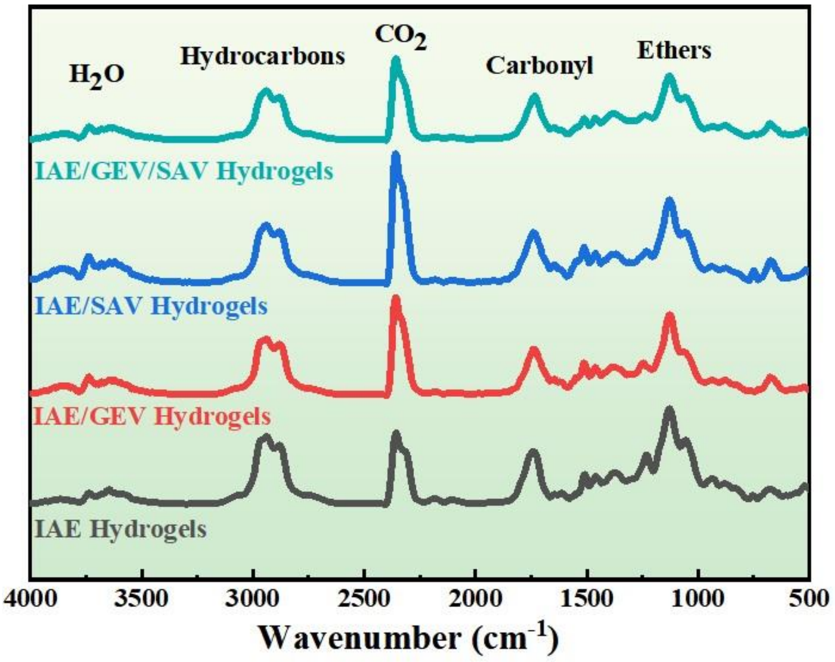

Figure S5. FTIR spectra of the pyrolysis products of all hydrogels at T_{max}.

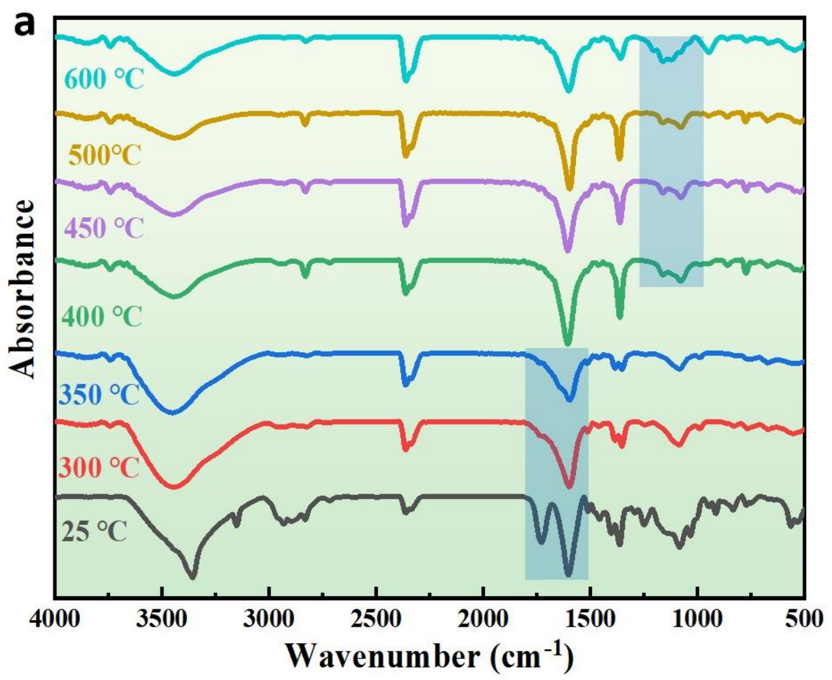
a
600 °C
500°C
450 °C
400 °C
350 °C
300 °C
25 °C
Absorbance
4000 3500 3000 2500 2000 1500 1000 500
Wavenumber (cm-1)

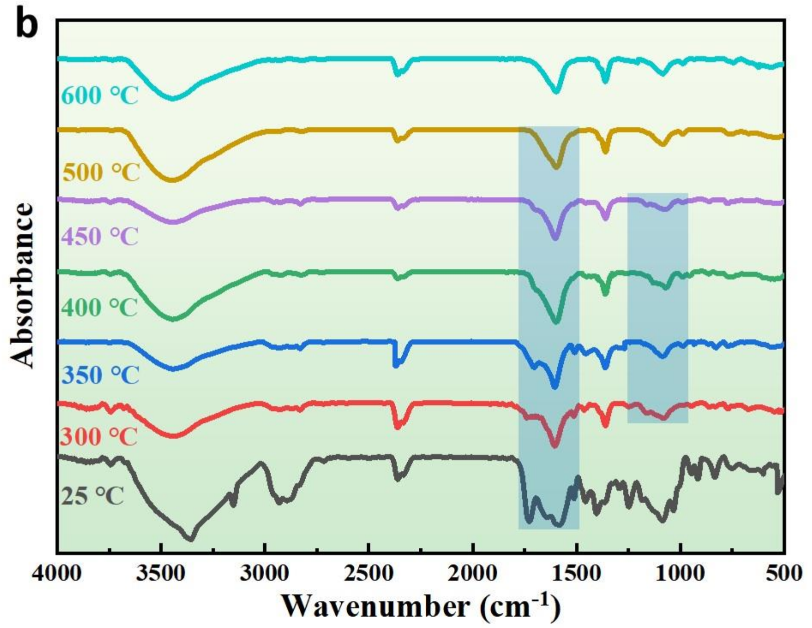
b
600 °C
500 °C
450 °C
400 °C
350 °C
300 °C
25 °C
Absorbance
4000 3500 3000 2500 2000 1500 1000 500
Wavenumber (cm-1)

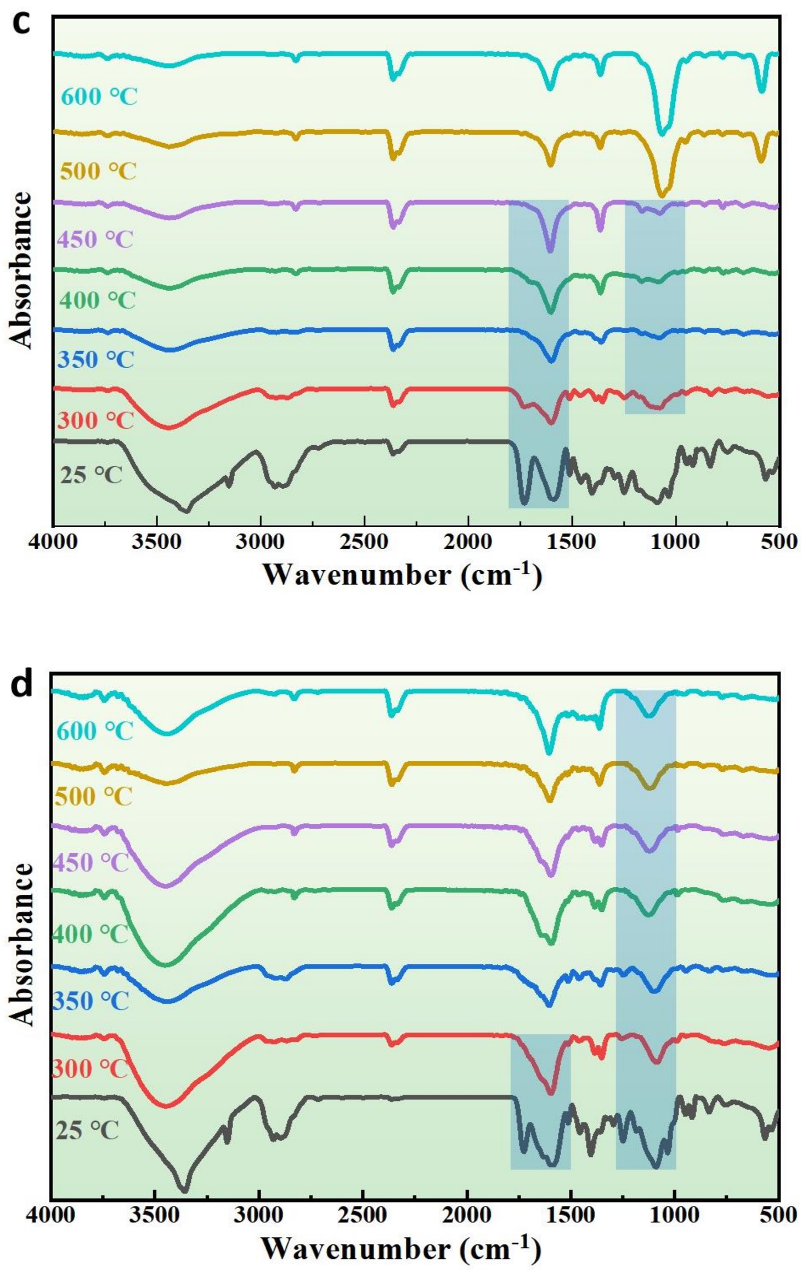

Figure S6. FTIR spectra of the condensed products of (a) IAE hydrogels, (b) IAE/GEV hydrogels, (c) IAE/SAV hydrogels and (d) IAE/GEV/SAV hydrogels at different temperatures.

Table S4. Stress values for 3D printed hydrogels and 405 nm LED cured hydrogels.

Stress (KPa)	3D printed hydrogels	LED cured hydrogels
IAE hydrogels	98.0 $\pm$ 3.69	26.2 $\pm$ 4.86
IAE/GEV hydrogels	114.5 $\pm$ 4.63	65.1 $\pm$ 6.08
IAE/SAV hydrogels	144.3 $\pm$ 5.05	76.59 $\pm$ 5.18
IAE/GEV/SAV hydrogels	197.1 $\pm$ 5.23	99.83 $\pm$ 7.71

REFERENCES

[1] Zuo, X. L.; Wang, S. F.; Zhou, Y.; Wu, C.; Huang, A. R.; Wang, T. F.; Yang, Y. Y. Fluorescent hydrogel actuators with simultaneous morphing- and color/brightness-changes enabled by light-activated 3D printing. *Chem. Eng. J.*, **2022**, *447*, 137492.

[2] Zuo, X. L.; Zhang, K. Z.; Lei, Y.; Qin, S. H.; Hao, Z.; Guo, J. B. Influence of thermooxidative aging on the static and dynamic mechanical properties of long-glass-fiber-reinforced polyamide 6 composites. *J. Appl. Polym. Sci*., **2014**, *131*, 39594.

[3] Zuo, X. L.; Song, H. S.; Shao, H. J.; Aud, M. H.; Wei, T.; Guo, J. B. Effects of OMMT on the aging behaviors of halogen-antimony flameretarded LGFPA6 composites: Flammability and thermal degradation. *Thermochim. Acta*., **2017**, *653*, 32-42.

[4] Zuo, X. L.; Shao, H. J.; Zhang, D. H.; Hao, Z.; Guo, J. B. Effects of thermal-oxidative aging on the flammability and thermaloxidative degradation kinetics of tris(tribromophenyl) cyanurate flame retardant PA6/LGF composites. *Polym. Degrad. Stabil*., **2013**, *98*, 2774-2783.

[5] Liu, J. R.; Yu, Z. C.; He, H. L.; Wang, Y. S.; Zhao, Y. H. A novel flame-retardant composite material based on calcium alginate/poly (vinyl alcohol)/graphite hydrogel: thermal kinetics, combustion behavior and thermal insulation performance. *Cellulose*. **2021**, *28*, 8751-8769.

[6] Xua, S. S.; Liang, W. C.; Xu, G. Z.; Huang, C. J.; Zhang, J. Y.; Lang, M. D. A fast and dual crosslinking hydrogel based on vinyl ether sodium alginate. *Appl. Surf. Sci*., **2020**, *515*, 145811.
